# A Neuroanatomy of Positive Affect Display – Subcortical Fiber Pathways Relevant for Initiation and Modulation of Smiling and Laughing

**DOI:** 10.3389/fnbeh.2022.817554

**Published:** 2022-03-30

**Authors:** Volker A. Coenen, Bastian E. A. Sajonz, Trevor A. Hurwitz, Marlies Böck, Jonas A. Hosp, Peter C. Reinacher, Horst Urbach, Ganna Blazhenets, Philipp T. Meyer, Marco Reisert

**Affiliations:** ^1^Department of Stereotactic and Functional Neurosurgery, Faculty of Medicine, University Medical Center Freiburg, University of Freiburg, Freiburg, Germany; ^2^Center for Deep Brain Stimulation, University of Freiburg, Freiburg, Germany; ^3^Department of Psychiatry, University of British Columbia, Vancouver, BC, Canada; ^4^Department of Neurology and Clinical Neuroscience, Faculty of Medicine, University Medical Center Freiburg, University of Freiburg, Freiburg, Germany; ^5^Institute for Laser Technology (ILT), Aachen, Germany; ^6^Department of Neuroradiology, Medical Faculty, University Medical Center Freiburg, University of Freiburg, Freiburg, Germany; ^7^Department of Nuclear Medicine, Faculty of Medicine, University Medical Center Freiburg, University of Freiburg, Freiburg, Germany

**Keywords:** deep brain stimulation, essential tremor, laughter, motorMFB, pathological laughter, pseudobulbar affect

## Abstract

**Background:**

We here report two cases of stimulation induced pathological laughter (PL) under thalamic deep brain stimulation (DBS) for essential tremor and interpret the effects based on a modified neuroanatomy of positive affect display (PAD).

**Objective/Hypothesis:**

The hitherto existing neuroanatomy of PAD can be augmented with recently described parts of the motor medial forebrain bundle (motorMFB). We speculate that a co-stimulation of parts of this fiber structure might lead to a non-volitional modulation of PAD resulting in PL.

**Methods:**

We describe the clinical and individual imaging workup and combine the interpretation with normative diffusion tensor imaging (DTI)-tractography descriptions of motor connections of the ventral tegmental area (VTA) (*n* = 200 subjects, HCP cohort), [[^18^F] fluorodeoxyglucose (^18^FDG)] positron emission tomography (PET), and volume of activated tissue simulations. We integrate these results with literature concerning PAD and the neuroanatomy of smiling and laughing.

**Results:**

DBS electrodes bilaterally co-localized with the MB-pathway (“limiter pathway”). The FDG PET activation pattern allowed to explain pathological PAD. A conceptual revised neuroanatomy of PAD is described.

**Conclusion:**

Eliciting pathological PAD through chronic thalamic DBS is a new finding and has previously not been reported. PAD is evolution driven, hard wired to the brain and realized over previously described branches of the motorMFB. A major relay region is the VTA/mammillary body complex. PAD physiologically undergoes conscious modulation mainly *via* the MB branch of the motorMFB (limiter). This limiter in our cases is bilaterally disturbed through DBS. The here described anatomy adds to a previously described framework of neuroanatomy of laughter and humor.

*‘Against the assault of laughter, nothing can stand.’*

— Mark Twain

## Introduction

Presentation of positive affect – smiling and laughing – is important for interindividual signaling between primates. Both, smile and laughter have important functions in human social context and the amount of facial activity during smiling – especially presentation of Duchenne smiles – is interpreted as the degree of trustworthiness, cooperation, openness, and sociability (Parkinson, 2005; Mehu et al., 2007). We will here sum up both expressions as a continuum under the term “positive affect display” (PAD).

With the important functions of PAD (emotional, social motive, personal, and communication) for the human primate in mind it seems conceivable to assume specialized anatomy in humans and such systems could potentially be visualized with modern imaging technologies. While the idea of a neuroanatomy for PAD is not entirely new (Parvizi et al., 2001; Wild et al., 2003) the existing descriptions typically focused on lesion models or electrical stimulation experiments without associated sophisticated imaging and certain subcortical pathways have been suspected (Wilson, 1924) but to this day are elusive in these descriptions.

We have here analyzed the cases of two patients (66 f/70 m) with thalamic deep brain stimulation (DBS) for essential tremor (ET) (Vim, ventral intermediate nucleus) who during the course of successful tremor-ameliorating stimulation reacted with distinct forms of pathological laughter (PL). We present some new evidence for the existence of a previously described system of neuronal subcortical pathways (Hosp et al., 2019) [motor medial forebrain bundle (motorMFB)] – projections involving the ventral tegmental area (VTA), mammillary bodies, brainstem, cerebellum, motor cortices, and the prefrontal cortices – which might facilitate PAD.

## Materials and Methods

### Case Descriptions

#### Case #1

A 66 year/old female received DBS electrodes (model 3389, Medtronic, United States) and an ACTIVA PC system (Medtronic, United States) for her therapy refractory ET including a no–no head tremor. The ventral intermediate nucleus of the thalamus (Vim) was targeted without diffusion tensor imaging (DTI) tractographic assistance with a stereotactic frame. Macro-testing showed excellent improvement intra-operatively and on follow-up [3/3 tremor improvement, full tremor control according to a previously published simplified rating scale (Coenen et al., 2020a)]. In the weeks after initiation of stimulation the patient presented to the outpatient clinic with newly occurred episodes of pathological “mirthless laughter” (10–12 episodes per day, lasting seconds to minutes), typically elicited during situations with *tense* emotional content. A manic episode was excluded. These situations occurred with high frequency and were disturbing to her and leading to social isolation. Burst of laughter never led to enhanced mood or a reversal of the feeling of sadness. On the other hand, her sense of humor was never compromised and actually laughing during humorous situations was subjectively somewhat enhanced. She showed a tendency to smile and giggle somewhat inappropriately during outpatient visits while she herself described that internally she felt an emotional incongruence (feeling of tension, nervousness) regarding this behavior. She was previously diagnosed with episodic depression (unipolar) which at the time of surgery had not been treated with medication. Reduction to only unilateral DBS (either right or left) over days silenced the effect, and only bilateral stimulation elicited PL. Leaving one sided stimulation off was tested 2–3 times in the course of the disease (Neurology). The patient noticed the shutting off of DBS with contralateral lack of tremor control (no blinding possible). Stimulation was stopped at nighttime (as typical in ET) (Kronenbuerger et al., 2006). Uncontrolled laughing only occurred under *bilateral* stimulation (which she needed for her head tremor) and typically when confronted with an *emotional content (reported stress or nervousness)*. Stimulation parameters: Case +, 1-, 3.1 V, 60 us, 180 Hz; Case +, 8-, 2.6 V, 60 us, 180 Hz. Effective electrode positions were typical for the Vim target (Table 1). An antidepressant medication with Venlafaxine (Sandoz, Basel, Switzerland) was started and the frequency and severity of laughing attacks diminished. Because of a swollen tongue she later stopped this medication with some re-occurrence of laughter episodes but also of her depression.

**TABLE 1:** Coordinates of effective contacts during chronic stimulation.

			Left				Right			
Case	Space	ACPC length	*x*	*y*	*z*	PC	*x*	*y*	*z*	PC
#1	MNI	dna	−10	−19	−3	dna	12	−19.5	−2.5	dna
	MNI/MCP	25.5	−10	−7.5	2.5	5	12	−8	0	5
	Native/MCP	25.5	−12.5	−5.5	3	7	14	−7	1.0	5.5
#2	MNI	dna	−10	−20.5	1.5	dna	11	−19.5	3	dna
	MNI/MCP	25.5	−9	−9	3	4	11	−9	4.5	5
	Native/MCP	24	−11.5	−6	−3.5	6	(12)*	(5.5)*	(−3.5)*	(6.5)*

All values in mm; MNI, Montreal Neurological Institute Brain space; MCP, mid-commissural point coordinate system; PC, in front of PC; ()* = not chronically stimulated; negative z-axis coordinates (MCP) indicate stimulation “inferior” to the MCP plane. Case #2 differs from test stimulation since chronic stimulation occurred 1–2 contacts more inferior (and only unilaterally) because of better tremor effects.

#### Case #2

In a second case (70, m) DTI assisted thalamic DBS implantation (directional Cartesia leads and Vercise Gevia, Boston Scientific, Marlborough, MA, United States) was performed for bilateral ET. During test stimulation on the operation table the patient reacted to right-sided unilateral test stimulation (2 mA, 130 Hz, 60 us; 4 mm above target region) with a short burst of laughter (“*hahaha*”). He was not aware of any humorous content at the time and could not explain this behavior. He suffered from a small subcortical hemorrhage (posterior medial frontal gyrus, left) which gave him some transient word finding difficulties but fully resolved spontaneously without clinical sequelae. In the initial 2 months of unilateral stimulation (one contact deeper than intraoperative stimulation, because of better tremor improvement; C +, 1-, 2.7 mA, 60 us, 130 Hz) only to his left thalamus (at that time electrode lesioning effect still reduced left hand tremor) he reported improved tremor (improvement 3/3 for right hand, 2/3 for non-stimulated left hand) but also was told by his family that he continuously appeared more open to conversation and would smile more (accompanying video). The patient showed typical stimulation start paresthesias which resolved after about 10 s. There were no contractions. Later stimulation was performed at inferior contacts allowing for better tremor control in the course of the disease. After 13 months of unilateral left-sided stimulation (C +, 1-, 3.5 mA, 60 us, 130 Hz) he reports an overall improved mood. Also in this case effective electrode positions were typical for the Vim target (Table 1), although potentially slightly medial and posterior.

### Positron Emission Tomography-Imaging and Processing

Patient #1 received positron emission tomography (PET) imaging. Imaging was performed 10 months after initiation of DBS, once with DBS on and once with DBS off after DBS had been switched off for 72 h. PET scans were acquired on a Gemini TF 16 BigBore integrated PET/CT system (Philips, Netherlands) after the patient fasted for >6 h to ensure normal plasma glucose levels. After a low-dose CT for attenuation correction, a 10-min PET scans were started at 50 min after intravenous injection of 200 and 205 MBq [^18^F] fluorodeoxyglucose (FDG) under resting conditions (eyes open and ears unplugged at ambient noise). During each acquisition, the position of the patient’s head was gently restrained with an elastic tape and carefully monitored. Details of the PET processing and analysis can be found in Appendix.

### MR-Imaging/Image Workup

Both patients received preoperative MR imaging and a post-operative CT. The MR-measurements comprised anatomical scans (T1 and T2 weighted) and diffusion-weighted MRI (dMRI); details about the protocol are in Appendix. A post-operative CT was acquired to localize the DBS electrodes.

To relate individual anatomy to normative template information [Montreal Neurological Institute (MNI) space] we used the CAT12 toolbox. For diffusion MRI based tractography the global approach from Reisert et al. (2011) was used. The ICa/NAC and the Vim PET activations are used for streamline selection (Figures 2, 3). Due to the rather bad quality of the acquired dMRI data, we additionally relied on a normative HCP connectome to relate the stimulation site and PET activation to known white matter anatomy. Mainly, the motor associated projections of the VTA as described by our group in Hosp et al. (2019) are considered: one reaching the prefrontal cortex (PFC), the mammillary body (MB), and the brainstem/cerebellum (BC). These bundles were further constrained to run through the two PET activations (left ICa/NAC and Vim) and the volume of tissue activated (VAT) of the stimulation electrodes (Figures 2, 3). We further rendered the associated cortical projection in terms of streamline terminal densities on a cortical surface mesh (MNI-152 iCC template) (Figure 4). In addition, we also considered the dentato-rubro-thalamic tract (DRT) and the fornix (FC) (Figures 3, 5). To relate the found tract courses to the thalamic subnuclei, the atlas of Morel (2007) is taken into account (Figure 6). For more details about the processing steps, we refer to the Appendix.

## Results

The results are shown mainly in the figures and figure legends.

**FIGURE 1:**
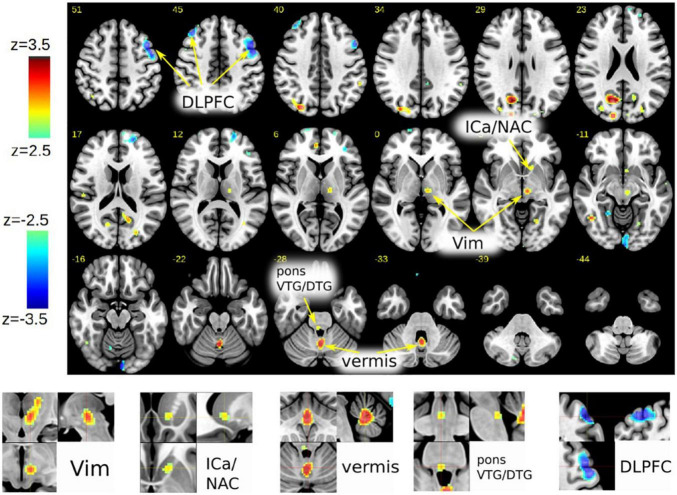
Case #1, FDG-PET activation pattern contrasting DBSon vs. DBSoff. Effects shown are related to initiation of DBS. Pattern description in text. (DLPFC, dorsolateral prefrontal cortex; ICa, anterior limb of internal capsule; NAC, nucleus accumbens; Vim, ventral intermediate nucleus of thalamus; VTG, ventral tegmental nucleus of Gudden; DTG, dorsal tegmental nucleus of Gudden.)

In case #1, FDG PET revealed a specific pattern of activation and deactivation in contrasting DBS_ON_ vs. DBS_OFF_. A left-sided activation (DBS_on_) of the thalamic region surrounding the left DBS electrode but clearly extending caudally into the VTA was observed. *Increased metabolism of left ICa and NAC were seen as an effect of DBS potentially activating the PFC and slMFB pathways*. Increased metabolism was noted in the cerebellar vermis and in the close to midline tegmental pons possibly congruent with the Gudden nuclei [ventral tegmental nucleus of Gudden (VTG) and dorsal tegmental nucleus of Gudden (DTG)]. There was a reduced metabolism bilaterally in the DLPFC (left > right) extending somewhat into the precentral region (M1) on the left. Occipital and parietal activations were noted which probably are non-specific (e.g., related to unintended minor differences in visual stimulation, artifacts) (Figure 1). Patient #2 did not receive PET imaging (refused).

**FIGURE 2:**
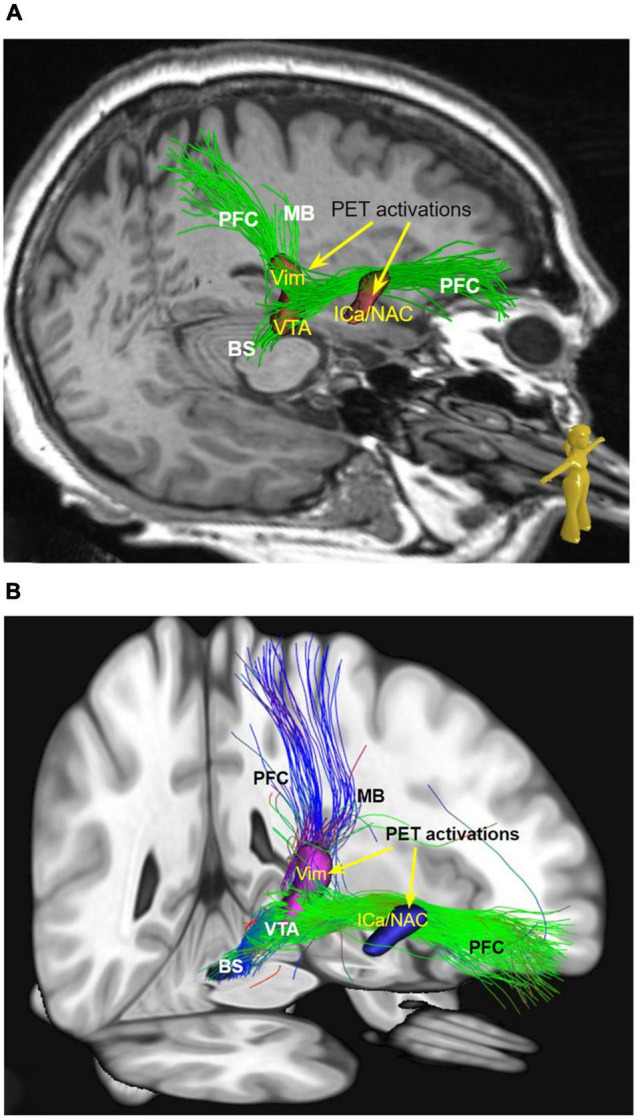
**(A)** Streamlines selected from global tractography based on the subject’s dMRI data (native space). **(B)** Same as a but in MNI space and normative dMRI data. Streamlines are constrained to visit the ICa/NAC and the Vim PET activation. MB pathway cannot be visualized in patient individual imaging but shows up on normative data. (MB, mamillary bundle of motorMFB; PFC, prefrontal cortex branch of motorMFB; Vim, ventral intermediate nucleus; VTA, ventral tegmental area; OFC, orbitofrontal cortex; ICa, internal capsule anterior limb; NAC, nucleus accumbens.)

**FIGURE 3:**
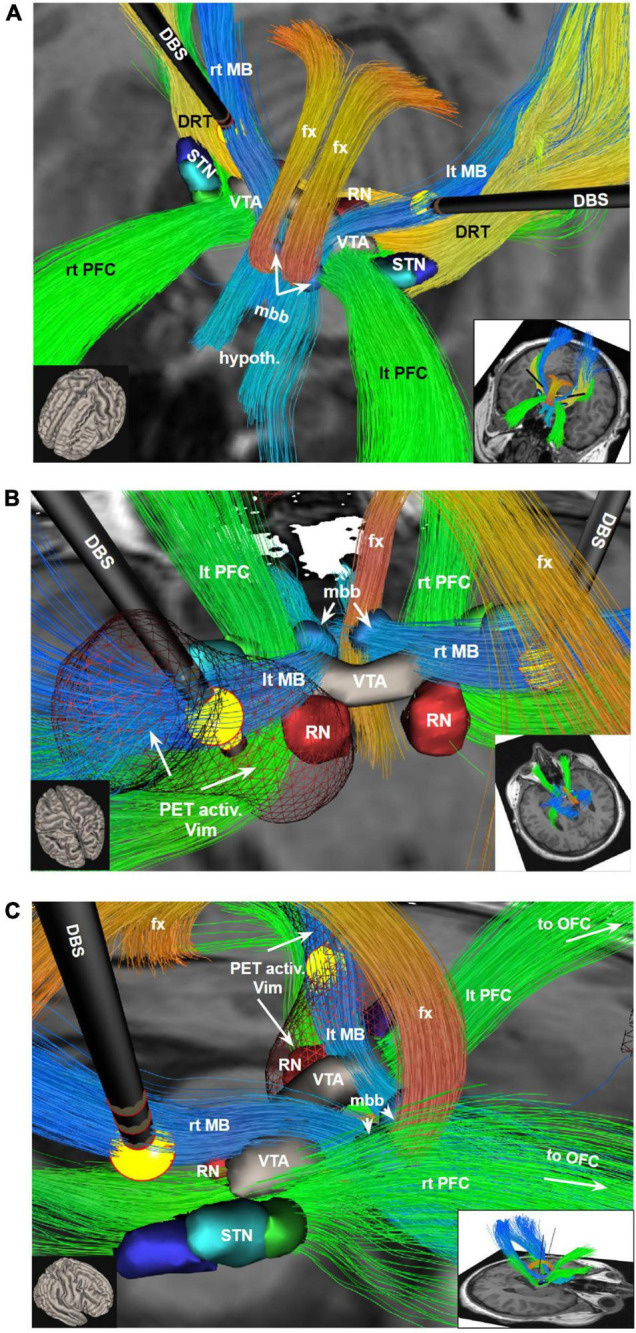
Bilateral DBS electrodes perfectly hit MB bundles just medial to the DRT. The bilateral stimulation effectively reduces tremor (*via* DRT, not shown) while at the same time modulating the limiter pathway (MB); **(A)** view from anterior and upper left; **(B)** view from posterior and upper left. PET activation surrounding the left DBS *electrode extends downward into the VTA and also encroaches on the PFC pathway (in PET imaging, Figure 1, leading to ICa/NAC activation)*. **(C)** View from right. To understand the relationship of the stimulation electrodes and the white matter geometry we show here bundle specific tractograms (based on normative connectome data from HCP) in the native space of the considered case. Additionally, deep GM structures are shown, which are partly taken from atlases, but were also manually drawn. DBS, deep brain stimulation electrode with volume of tissue activated in yellow; DRT, dentato-rubro-thalamic bundle; fx, fornix; hypoth., hypothalamus; lt, left; MB, mamillary body branch of motorMFB; mbb, mamillary body; OFC, orbitofrontal cortex; PET activ., PET activation, PFC, prefrontal cortex branch of motorMFB, RN, red nucleus; rt, right; STN, subthalamic nucleus; VTA, ventral tegmental area.

**FIGURE 4:**
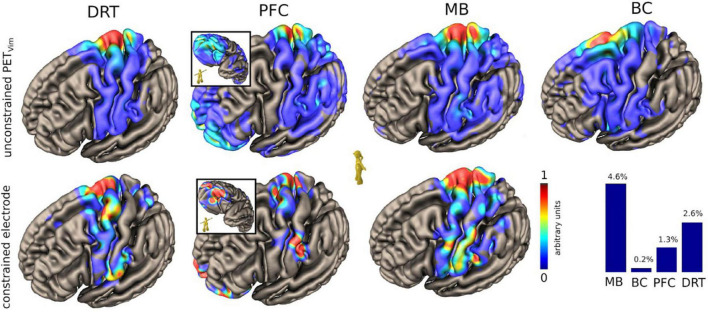
Case #1, unconstrained and constrained cortical projection patterns of the DRT and the regarded pathways relevant for PAD (HCP group level, MNI-space). *Upper panel shows cortical projection patterns constrained to thalamic (Vim) PET activation. Lower panel shows a pattern constrained to the actual volume of activated tissue of the DBS. Note how PFC and MB focus on the facial region of the sensory-motor cortex*. PFC branch cortical pattern shows central and lateral orbitofrontal involvement (inset). Lateral OFC is typically involved in response inhibition (Kringelbach, 2005). The lower right *histogram shows a simulated percentage of tract activation*. DRT, dentato-rubro-thalamic tract; PFC, MB and BC represent branches of motorMFB with distinct regions of origin: PFC, prefrontal cortex branch; MB, mamillary body branch; BC, brainstem cerebellum branch (see also Figure 7).

**FIGURE 5:**
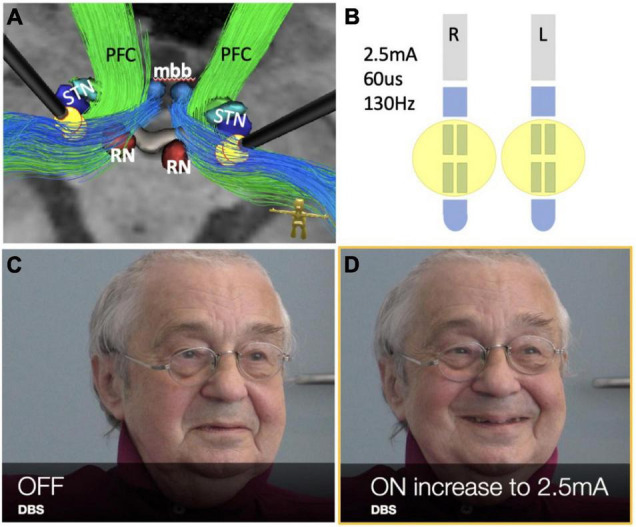
Case #2, bilateral stimulation in the MB tracts (limiter pathways) during thalamic DBS for tremor. See also the accompanying video. **(A)** DBS electrode positions with stimulation pattern as shown in **(B)**. **(B)** Stimulation pattern used to evoke PAD response in postoperative phase (**see also accompanying video**). **(C,D)** Note initiation of PAD *via* bilateral stimulation (and inhibition) of limiter pathways (blue in **A**). (RN, red nucleus; STN, subthalamic nucleus; mbb, mammillary bodies; PFC, PFC bundle.)

**FIGURE 6:**
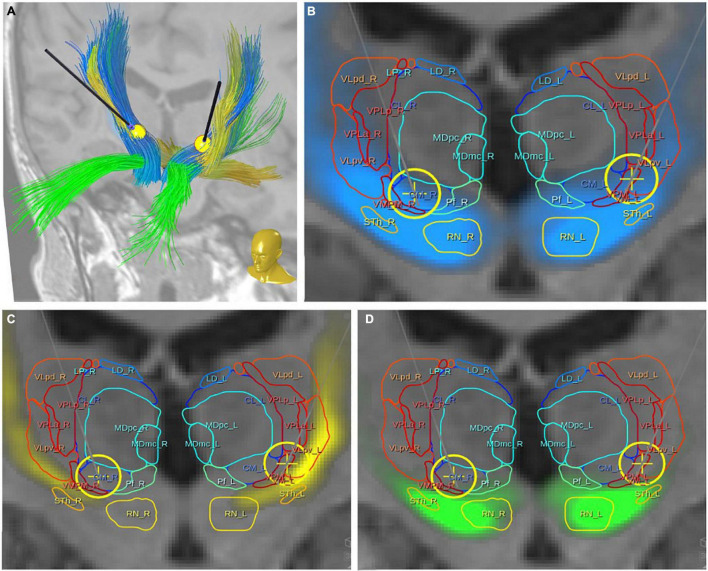
Case #1, topographic presentation of three fiber tracts with respect to thalamic nuclei [following the atlas of Morel (2007)]. **(A)** Overview with DBS electrodes showing three principle fiber pathways: DRT, yellow; PFC, green; MB, blue. **(B,C)** Relation of the regarded fiber tracts to thalamic nuclei and DBS electrodes with simulated volumes of activated tissue (yellow spheres). All three fiber tracts have proximity with the ventro-lateral thalamic nuclei [for nuclei abbreviations refer to Morel (2007)].

**Case #2**, the evaluation of the imaging data in this patient showed a similar involvement of the MB-pathway bilaterally (Figure 5) as seen in case #1. In order to investigate the “limiter pathway” theory – so the modulation of volitional PAD display *via* DBS – some weeks after surgery we performed an ON/OFF experiment in which we stimulated the middle segmented ring contacts of his DBS electrodes bilaterally (2–2.5 mA, 60 us, 130 Hz) which are known to target the MB branch (limiter pathway). The patient was aware of the experimental condition (switching on and off) but was only afterward made aware of the purpose (surveillance of PAD display). The interviewer was aware of the stimulation condition because the patient reported tingling of his hands on stimulus initiation (as seen in the video). Depending on the stimulation state, the facial display of his affect undulated in its magnitude (neutral ↔ smile ↔ laughter) (Figures 5C,D), despite unchanged mood during the session. As has been described by Hassler (1961), we also found a kindling phenomenon, indicating that a repeated stimulation of the pathway increases its response. The session was videotaped (*please also see supplemental video material including subtitles*).

## Discussion

We have analyzed two cases of stimulation induced PAD under bilateral thalamic DBS and found involvement of subcortical fiber structures. The observed phenomena meet the criteria of “*pathological laughter*.” In case #1 we found a specific PET activation/deactivation pattern (Figure 1) which coincided with a fiber pathway system (motorMFB) connected to the VTA and previously described by our group Figure 3 (Hosp et al., 2019). The same fiber pathway connection pattern was found in case #2 (Figure 5). In both cases the principle limiter (MB, limiter pathway) of emotional affect display – which in fact and under physiological conditions enables a volitional control of PAD – was disturbed through bilateral stimulation leading to PL.

Affect is an immediate (cross-sectional) emotion expressed through gesture, tone of voice or a facial configuration and accompanied by a congruent subjective experience. Darwin (1872) offered remarkable insights into the facial display of emotions and was the first to compare interspecies expression. The display of an emotional state helps to receive individual attention or care. Human evolution has introduced further functions, which are *enhancement of communication and the expression of social motives* and mimicry of positive facial affect – “smiling” – presentation is related to the feeling of affiliation and empathy (Drimalla et al., 2019). Laughing is complex and not well understood and has involuntary (volatile) and voluntary (controlled) features (Caruana et al., 2015). Emotional smiling and laughter are both involuntary and lean on an automated inherited multisynaptic circuit of coordinated musculature with sympathetic and parasympathetic (autonomic) connectivities (Provine, 1996, 2015). In dynamic social situations, laughing is contagious and *will precede the cognitive perception of humor*.

We follow a previously defined idea that smiling and laughter form a continuum where smiling can be regarded as a diminutive form of laughter (Mehu et al., 2007; Mehu and Dunbar, 2008). The theory that emotional expressions are inborn and directly hardwired to brain anatomy was first followed by Darwin (1872). Smiling and laughter appear to be determined motor circuits manifesting shortly after birth becoming increasingly sophisticated and adaptive by exposure to social contexts in conjunction with developing cognitive capacities (Davis and Montag, 2019). Children who are born blind can display positive affect (smile) while they for obvious reasons – cannot facially mimic such behavior (Freedman, 1964). Laughing (*and its opposite, crying*) appear to have specific and more important functions than the display of other moods which can be inferred from the mere existence of pathological states (Parvizi et al., 2001; Lauterbach et al., 2013) in which they are dysregulated (e.g., pseudobulbar affect, PL, etc.).

### Pathological Laughter

Both our patients presented with a distinctive form of PL (Mendez et al., 1999; Parvizi et al., 2006; Madani et al., 2013) which is a subclass of involuntary laughter characterized by the presence of episodic and contextually inappropriate or exaggerated outbursts of laughter without commensurate feelings. In most cases (as in *our case #1*) the expression of laughter is not accompanied by the experience of pleasure (affect incongruent) *but may* – as in *our case #2 – be affect* congruent and then an exaggeration of a normal response. Unilateral midbrain related focal lesions have been described as the origin of PL (Ron et al., 2004; Madani et al., 2013) and there have been multiple reports on the induction of smiling and laughter as inadvertent side effects of cortical and deep brain stimulation at distinctive stimulation sites (Krack et al., 2001; Haq et al., 2010, 2011; Okun et al., 2010; Caruana et al., 2015; Huang et al., 2018).

### Subcortical Fiber Pathways and the Neuroanatomy of Smiling and Laughter

Based on our observations and an improved understanding of neural fiber pathways and connectivities through modern technologies we propose a revised anatomy of the circuitry involved in the expression of laughter. This relies heavily on our characterization of the ***greater MFB system*** as a in part bidirectional limbic and motor projection pathway carrying neurotransmitter critical fibers from the brainstem to cortical and subcortical structures. As seen in case #1 involuntary laughter occurs only in the *bilateral* DBS on condition. The PET activation pattern reveals the putative mechanism. Bilateral DBS modulates bilateral PFC *via* inhibition of the MB (limiter) of the motorMFB.

The motorMFB (Hosp et al., 2019) connects regions of the PET activation pattern (case #1) during the activation state for the laughing phenomenon (DBS ON, bilaterally) and thereby allows further conclusions on the mechanisms of PAD. Parts of this motorMFB (esp. MB bundle) are affected bilaterally in both cases, despite the distinct magnitude of PAD, laughing attacks in case #1; smiling and some laughing in case #2. The MFB (human) is a complex system connecting brainstem and cerebellum with subcortical and cortical structures and funneled through a common pipeline, the VTA. The MFB is – more functionally than anatomically – related to the mfb [rodent (Nieuwenhuys et al., 1982; Veening et al., 1982; Geeraedts et al., 1990a,b)] and its trans-hypothalamic route (Coenen et al., 2009, 2011, 2021). The MFB – especially its superolateral branch, the slMFB – is confluent with major parts of the reward (SEEKING) system (Coenen et al., 2011, 2012, 2018b,2020b). The MFB regulates perception of appetitive or aversive responses and regulates motor activity toward appetitive or away from aversive triggers. As such, the motorMFB is closely related to the MFB system: in our interpretation, motor responses based on emotion valence evaluation and an according attentional shift are to be an expected function besides its role during motor learning (Hosp et al., 2019). In this respect, the facial presentation of PAD (smiling, laughing) fully falls into the remit of its function.

**FIGURE 7:**
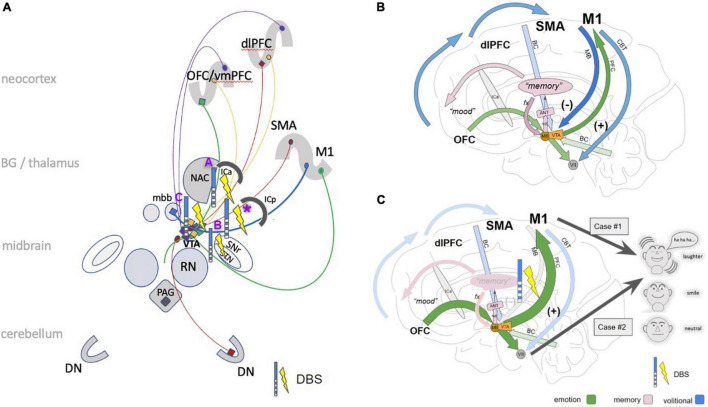
Cartoonistic rendition of subcortical pathways for PAD. **(A)** The simplified wiring pattern of the different pathways involved: MB (blue), =limiter-pathway, M1 to VTA/mbb complex inhibiting PAD; PFC pathway (green), connects OFC *via* VTA to M1 and brainstem; red, BC pathway (connects dlPFC *via* VTA to brainstem and cerebellum). imMFB (purple); connects VTA to OFC and dlPFC (dopaminergic); slMFB (yellow), bidirectional between OFC/dlPFC and VTA. Selected stimulation effects with pathological or induced laughter out of the literature: **(A)** stimulation in the anterior limb of the internal capsule (Okun et al., 2010; Haq et al., 2011); **(B)** subthalamic nucleus (STN) DBS (Krack et al., 2001); **(C)** slMFB DBS in major depression (Schlaepfer et al., 2013; Coenen et al., 2018a); *, this contribution. **(B)** In the proposed concept the PFC branch (green arrows) of the motorMFB [OFC to M1 and facial nuclei (vii)] serves to immediately display (positive) affect (smiling, laughing). It is regulated, depending on environmental needs via the mamillary body branch (MB, blue arrow) as a “limiter pathway” which inhibits PAD (at the level of mbb/VTA complex). **(C)** Modulation of MB pathway via DBS leads to a dysfunctional control and thereby to (pathological) laughter (case #1) or simply enhanced PAD (case #2). (dlPFC, dorsolateral prefrontal cortex; OFC, orbitofrontal cortex; vmPFC, ventromedial prefrontal cortex; SMA, supplementary motor area; M1, primary motor cortex; ICa, anterior limb of the internal capsule; mbb, mammillary bodies; PAG, periaqueductal gray; STN, subthalamic nucleus; VTA, ventral tegmental area (of Tsai); SNr, substantia nigra; RN, red nucleus; NAC, nucleus accumbens; DN, dentate nucleus; CBT, cerebro-bulbar tract; VII, nucleus of the facial nerve; ANT, anterior nucleus of thalamus, PAG, periaqueductal gray.)

Our results augment previous concepts of laughter and affect regulation in general (Parvizi et al., 2001; Meyer et al., 2007). In our novel hypothesis the cerebellum plays a critical role in the regulation of the intensity and duration of the motor expression of emotion similar to its role in other motor behaviors (Figures 7B,C). In healthy individuals, the cerebellum modulates the profile of emotional responses unconsciously and automatically according to the information it receives from the cerebral cortex regarding the cognitive and social context of the triggering stimulus. When – functionally – disconnected from the cerebral cortex, the deafferented cerebellum produces exaggerated or contextually inappropriate responses. The traditional prior hypothesis was loss of voluntary control of facio-respiratory circuitry located in the brain stem from bilateral lesions of descending corticobulbar tracts (Damasio et al., 2000; Parvizi et al., 2001, 2006). Our VTA pipeline carrying MFB projection fibers between brainstem and telencephalic structures are consistent with both hypotheses and might help to reconcile them. The slMFB branch of the MFB serves as the involuntary pathway connecting the OFC with the brainstem (especially facial nuclei) while the PFC and MB bundles of the motorMFB connect brainstem to motor and prefrontal cortices (Hosp et al., 2019; Figure 7).

Positive affect display is elicited upon modulation of the PFC and MB bundles. These pathways run in proximity to the subthalamic nucleus (Figures 3, 7) and are potentially the very pathways which are activated in affect state induction during DBS (Krack et al., 2001; Haq et al., 2010, 2011) at different anatomical points as also demonstrated by our PET data. Moreover, the co-activation of the slMFB might be responsible for induction of mirth, producing affect-congruent smiling (Schlaepfer et al., 2013) and laughter. Laughter has been noted during deep brain stimulation in the anterior limb of the internal capsule (ICa, ALIC target) (Okun et al., 2010; Haq et al., 2011) which has been shown to predict a long term treatment efficacy to vc/vs DBS in the treatment of OCD (Haq et al., 2011). Our PET data provides confirmatory data where the DBS on state produced activation in the vc/vs region (Figure 1). Parvizi has named the ventral striatum and vmPFC amongst other structures as *emotion induction sites* (Parvizi et al., 2001). These responses have been attributed to the activation of reward and reward related learning circuitry. In our DBS case #1 we found co-modulation of the PFC and MB bundles of the motorMFB but the cortical effect was inhibitory (Figure 1). The MB bundle which we have labeled as the *limiter pathway* connects to M1 (Figure 7). DBS inhibition of the motor cortex may result in bilateral corticobulbar deafferentiation and thus supports the traditional hypothesis of loss of voluntary control of facio-respiratory circuitry located in the brain stem from bilateral lesions of descending corticobulbar tracts (Parvizi et al., 2001, 2006). It is in this respect not unusual to think about the existence of cortico-cortical short circuit connections (Catani et al., 2012) as direct regulators of cortical function. In turn the MB-bundle is the anatomical realization of the inhibiting cortico-bulbar connection that has been anticipated (Wilson, 1924; Parvizi et al., 2001). The MB bundle connects to the mamillary bodies and therefore with Papez’ episodic memory-circuit (Papez, 1937; Figure 7). Thus, memory provides the context in which current events occur and context appreciation provides the background tension in which events are experienced as humorous.

As seen in our PET data, rostral activation of the BC bundle produces PFC and M1 inhibition. This bundle also has a caudal trajectory and connects with ipsilateral cerebellum and brainstem (Hosp et al., 2019). The brainstem contains multiple nuclei that form part of the limbic system. Amongst these only the VTG and DTG both lying dorsally in the pons connect to the mammillary bodies (mbb). These nuclei reach the mbb by way of the mammillotegmental tract and mammillary peduncle exiting the midbrain *via* the VTA and are likely activated with stimulation of the BC bundle. Such activation is seen in our PET data (Figure 1). Also as can be seen from our PET data BC bundle stimulation activates the cerebellar vermis which is responsible for coordination of movements of the central body and would be the prime cerebellar anatomical candidate to control the faciorespiratory circuitry responsible for the expression of emotion. Simultaneous activation of the cerebellar vermis and deactivation of motor and PFC cortices fits the updated anatomical model for PL in which cerebellar dysregulation is the central disturbance caused by disconnection from the cerebral cortex. In this circumstance the cerebellum is no longer receiving appropriate cognitive and social feedback producing exaggerated or contextually inappropriate responses (Damasio et al., 2000; Parvizi et al., 2001, 2006). Our data thus reconcile the two hypotheses that explain the emergence of pathological PAD-bilateral corticobulbar deafferentation and cerebellar dysregulation.

### Unresolved Issues

#### Why Do We Not See Positive Affect Display More Often Under Thalamic Deep Brain Stimulation?

Hassler (1961) reported initiation of smiling in 9% and fully initiated PL in 4% of thalamic ventral and lateral thalamic (Vo) stimulation cases. With such high incidence during thalamic DBS cases, it might be that intraoperatively such responses are simply overlooked. Another explanation might be patients’ idiosyncrasies; patients who suffer from depression might have a dysregulation of affect display. There is evidence that they are apprehensive about the experience of and show a tendency to suppress positive experiences (Werner-Seidler et al., 2013). In this respect it appears a possibility that especially patients with a history of depression might typically use the limiter pathways (BC) to control the experience of PAD with dramatic release of laughter upon dysfunction. This would also explain why an antidepressant medication alleviates PL under DBS (like in case #1). Another possibility is that laughter bursts represent episodes of focal mania but especially in witnessed situations this was clinically in both patients not the case.

#### Is Bilateral Stimulation Always Necessary to Elicit Positive Affect Display?

Pathological laughter has been reported with unilateral thalamic stimulation on the OR table before (Hassler, 1961). In our case #2, it also occurred intra-operatively with only right-sided stimulation but only as a brief episode of a laughter burst. It is interesting that this patient in the long run of unilateral but left-sided stimulation (>1 year) had no episodes of PL but showed an improved mood. We thus cannot say if the phenomenon would occur with only right-sided stimulation. During testing (cf. accompanying video) bilateral stimulation was applied weeks after surgery and the patient showed a clear stimulation induced PAD. At this time there might additionally have still been some lesioning effect explaining the effect. PAD is typically symmetrical and bilateral as a phenomenon. Thus, it appears thinkable, that there is also a necessary bilateral regulation/control. Okun et al. (2010) have seen unilateral phenomena and elicited contralateral smiles during DBS in the anterior limb of the internal capsule and nucleus accumbens in one patient. In a later series they replicated this effect and were able to induce contralateral smiles and then laughter, the latter at a much higher stimulation amplitude (Haq et al., 2011). With respect to our model we speculate that they have stimulated the PFC pathway and unleashed a laughter response in the VTA–MBB complex, overriding the limiter pathways. Our cases only partly replicate this situation and show NAC and ICa (case #1, PET, Figure 1) activation but a clear involvement of the MB-limiter-pathway, necessary for regulating the motor response. This pathway in our cases needed to be implicated bilaterally and we think that for a long-standing effect this needs to be the case. Potentially, this is the reason why this phenomenon is so rare.

### Limitations

The pattern of PAD activation and the anatomy described here is the result of the description of two cases of PL, induced by bilateral DBS of the thalamic region. Limitations might arise since a whole new anatomical interpretation is presented as the result of the evaluation of only two cases. It is moreover conceivable that the pathways described might likewise serve for the display of negative affect, but this has not been observed in the context of our case description. The accompanying video of patient #2 – showing the test condition – was not taped under blinded condition. Therefore the results must be interpreted with caution. However, it is impossible to blind patient and investigator for stimulation if this at the same time induces paresthesia and tremor alleviation which is typical for Vim DBS.

## Conclusion

In two cases of pathological PAD induced by thalamic DBS for ET we found an explanation in dysregulation of the extended MFB system. Under normal physiology PAD is dominantly non-voluntary – especially in its initiation – and originates in PFC from cognitive appraisal of environmental stimuli within a social context and modulated *via* the MB branches of the motorMFB (the limiter pathway). This latter pathway has been suspected to exist (Wilson, 1924) and is an inhibitory part of the corticobulbar pathways. A context appropriate fully expressed and experienced positive affect requires participation of memory related circuitry (Papez) to establish the contextual tension (MB bundle of the MFB) as well as positive affective valencing from coactivation of nucleus accumbens septi, the nuclear source of the emotion of pleasure (slMFB). A major relay region appears to be the VTA/mammillary body complex. The fundamental neural mechanisms for PAD appear to reside in a genetically determined multisynaptic circuit involving brainstem, cerebellum and cortical motor regions. The circuitry for conscious control appears to reside in dIPFC and the BC branch of the motorMFB. In the event of PL – in our cases through bilateral DBS – voluntary control over the PAD circuit is lost. The results from the investigations of our two cases are able to unify two current hypothetical mechanisms – bilateral corticobulbar deafferentation and cerebellar dysregulation by identifying the central role of the extended MFB. *To our knowledge, this is the first report of pathological PAD being evoked through chronic DBS in the ventral thalamus*.

## Disclosure

*Unrelated*: Coenen receives an ongoing collaborative grant from BrainLab (Munich, Germany). He is a consultant for Ceregate (Hamburg, Germany), Cortec (Freiburg, Germany), and Inbrain (Barcelona, Spain). Coenen has ongoing IITs with Medtronic (United States) and Boston Scientific (United States). Sajonz receives a research grant from Ceregate (Hamburg, Germany) unrelated to this publication. Reinacher receives research support from: Else Kröner-Fresenius Foundation (Germany) and Fraunhofer Foundation (Germany). He is a consultant for Boston Scientific (United States), Inomed (Germany), and BrainLab (Germany). The remaining authors have nothing to disclose.

## Data Availability Statement

The original contributions presented in the study are included in the article/supplementary material, further inquiries can be directed to the corresponding author.

## Ethics Statement

Ethical review and approval was not required for the study on human participants in accordance with the local legislation and institutional requirements. The patients/participants provided their written informed consent to participate in this study. Written informed consent was obtained from the individual(s) for the publication of any potentially identifiable images or data included in this article.

## Author Contributions

VC and MR: conceptualization and data interpretation. MR, VC, BS, and TH: data analysis (MRI, CT) and manuscript writing. GB and PM: data analysis (PET). MB, JH, PR, HU, GB, and PM: proofreading. All authors contributed to the article and approved the submitted version.

## Conflict of Interest

The authors declare that the research was conducted in the absence of any commercial or financial relationships that could be construed as a potential conflict of interest.

## Publisher’s Note

All claims expressed in this article are solely those of the authors and do not necessarily represent those of their affiliated organizations, or those of the publisher, the editors and the reviewers. Any product that may be evaluated in this article, or claim that may be made by its manufacturer, is not guaranteed or endorsed by the publisher.

## Figures and Tables

**FIGURE A1:**
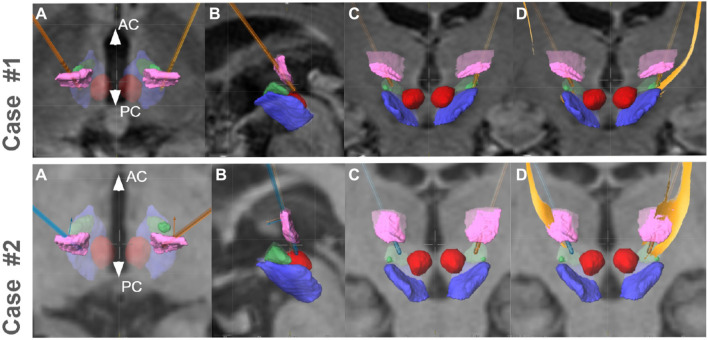
Implantation situation in both cases (upper panel, case 1, lower panel case 2). Ventral intermediate nucleus (Vim, pink) is traversed in both cases. Implantation occurred with the tips of the electrodes somewhat below the ACPC level (typical for Essential Tremor). **(A–C)** Axial–sagittal–coronal (respectively); **(D)** coronal with DRT reconstruction (yellow) from planning station (Elements, BrainLab, Munich, Germany). Other objects: red nucleus, red; subthalamic nucleus, green; substantia nigra, blue.

## Appendix

### Positron Emission Tomography Processing

Data sets were reconstructed using the LOR-RAMLA (line of response-row action maximum likelihood expectation algorithm) 3D iterative reconstruction algorithm (i.e., default brain reconstruction method; number of iterations = 2, number of subsets = 33, smoothing level = normal, resulting voxel size = 2.0 mm × 2.0 mm × 2.0 mm). All corrections (attenuation, scatter, randoms, normalization, and calibration) were applied during iterative reconstruction.

Positron emission tomography analysis was performed in analogy to a well-established routine for single-subject analysis of ictal and interictal single-photon emission computed tomography examinations (O’Brien et al., 2000) with the exception that the patient’s scans were spatially normalized not the individual’s MRI but to an in-house FDG PET template in Montreal Neurological Institute (MNI) space for further analyses. All processing steps were implemented with an in-house written pipeline in MATLAB R2019a (The MathWorks Inc., Natick, MA, United States) using Statistical Parametric Mapping (SPM) 12 software routines (www.fil.ion.ac.uk/spm): the DBS-on scan was co-registered to the DBS-off scan by affine registration. Then the latter was spatially normalized to the in-house FDG PET template in MNI space and the estimated spatial normalization parameters were applied to both scans. Both scans were normalized for their global counts (proportional scaling) using a threshold-based brain mask (35% of image maximum). Finally, a difference mask was calculated, smoothed with a 10-mm (full width at half maximum) Gaussian filter, *Z*-transformed (across all voxels) and threshold at | *Z*| > 2.5 (corresponding to a two-tailed *p*-value < 0.012). As described below, clusters of significant metabolic in- and decrease were overlaid onto various MRI datasets.

### MR-Imaging and Processing

The MR-measurements were acquired on a Siemens 3T TIM Trio (case #1) and a PRISMA (case #2), respectively. The diffusion-weighted MRI used an SE EPI sequence, with a TE of 95 ms and a TR of 8.5 s. The whole brain was covered with contiguous 2 mm slices with an in-plane resolution of 2 mm × 2 mm. The diffusion encoding was performed in 61 directions with an effective *b*-value of 1000 s/mm^2^. Additionally, a conventional *T1* data set (TR = 1390 ms, TE = 2.15 ms, 1 mm iso.) was acquired and a FLAIR (TE = 388 ms, TI = 1800 ms, TR = 5000 ms, 1 mm iso.). Further, a post-operative CT was acquired to determine the electrode location, which was co-registered *via* SPM (http://www.fil.ion.ucl.ac.uk/spm/software/spm12) to the anatomical MR data.

To map the patient space to MNI template space CAT12 (http://dbm.neuro.uni-jena.de/cat12/CAT12-Manual.pdf) implemented in the Statistical Parametric Mapping software (SPM12) was used. Tractography was performed using a global approach [6], which is implemented in a publicly available toolbox Diffuion&Fibertools toolbox (http://www.uniklinik-freiburg.de/mr-en/research_groups/diffperf/fibertools.html). For global tractography the toolbox provides two standard parameter sets, where we used the dense setting. As a reconstruction area the white matter segmentation obtained from CAT12 at the loose threshold of 0.1 was used to ascertain the inclusion of the subcortical gray matter areas. To understand the involved streamlines, the corresponding PET activations (left ICa/NAC and Vim) were used for selection.

Due to the rather bad quality of the acquired dMRI data, in particular in the midbrain area, where the susceptibility induced distortions are strong, we additionally relied on normative connectome information to relate the stimulation site and PET activation to white matter anatomy. As the basis for the group connectome 200 subjects from the HCP cohort are used (https://ida.loni.usc.edu/login.jsp) using the Q1: S3, S4 subsample (*n* = 200 subjects; 78 male; mean age ± SD, 29 ± 3.5 years). We followed the same strategy as described in Hosp et al. (2019): streamlines are selected in native subject space, streamline/terminal density are rendered into a volumetric representation and warped into group space to obtain a group average. As an extension to Hosp et al. (2019) we also generated directional fiber density maps to apply bundle specific tractography (Rheault et al., 2019). The directional fiber density maps were obtained by rendering the rank-1 tensor formed by the tangent of the selected streamlines in native space. The tensor field representation allows us to compute means in the common additive manner as for the scalar densities. However, the tensorial nature of the field has to be taken into account during warp to MNI standard space. Therefore, the rotational part of the Jacobian matrix of the associated template warp is used to rotate the local fiber directions. The obtained group representations of the directional maps are finally transformed into the native patient space in the same manner.

Mainly, the motor associated projections of the ventral tegmental area (VTA) as described by our group in Hosp et al. (2019) are considered. These projections are subdivided in Hosp et al. (2019) into three sub-bundles: one reaching the prefrontal cortex (PFC), the mammillary body (MB), and the brainstem/cerebellum (BC). Here, these bundles were further constrained to run through the two aforementioned PET activations (left ICa/NAC and Vim) and the volume of tissue activated (VAT) by the stimulation electrodes of the considered case. Therefore, the PET activations and the VATs had to be transformed into MNI space to streamline selection of the HCP cohort.

We further rendered the associated cortical projection in terms of streamline terminal densities on a cortical surface mesh (MNI-152 iCC template). In addition to the motor-related VTA projections, we also considered the dentato-rubro-thalamic tract (DRT) and the fornix (fx). The DRT was selected by three spherical ROIS, which were defined by the MNI coordinates p1 = (17, −58, −29), p2 = (8.5, −40, −30), and p3 = (−18.5, −16, 5) with radii r1 = 13, r2 = 4.5, and r3 = 5. The fornix was also selected by three spherical ROIS with coordinates p1 = (0, 2, 7), p2 = (0, −6, 14), and p3 = (0, −4, −13) with associated radii r1 = 4, r2 = 4, and r3 = 5.

The obtained tensor fields in patient space were used for deterministic, bundle specific tractography by using NORA’s bundle specific tractography as done in Coenen et al. (2020b). For most of the bundles they were obtained by randomly placing seeds in high density regions (threshold > 10^–1^) with a very loose stopping criterion (threshold > 10^–5^). For the MB bundle and the FC bundles the seeds were specifically placed in the mammillary body. The definitions of the nuclei were partly taken from publicly available atlases but were also drawn manually. Definitions of the red nucleus were based on (Ewert et al., 2018); definitions of the sub thalamic nucleus and its subdivision is taken from Ilinsky et al. (2018). The VTA and the mamillary bodies were manually delineated by an experienced neuroscientist based on the T1 and T2 weighted MR images.

To relate the found tract courses to the thalamic subnuclei, the atlas of Morel (2007) is transformed from MNI space into the subject space of case #1.

## Abbreviations

^18^FDG: [^18^F] fluorodeoxyglucose
ALIC: anterior limb of internal capsule (DBS target)
BC: PFC to brainstem/cerebellum (mtrMFB)
DBS: deep brain stimulation
ICa: anterior limb of the internal capsule (anatomical)
ICp: posterior limb of the internal capsule (anatomical)
dlPFC: dorsolateral PFC
DTG: dorsal tegmental nucleus of Gudden
DTI: diffusion tensor imaging
fx: fornix
MB, bundle: M1 to mammillary body (mtrMFB), “*limiter pathway*”
mbb: mammillary bodies
MD: major depression
mfb: medial forebrain bundle (rodent)
MFB, medial forebrain bundle (system: human)
MRI: magnetic resonance imaging
mtrMFB: motorMFB
NAC: nucleus accumbens septi
OCD: obsessive–compulsive disorder
OFC: orbitofrontal cortex
PAD: positive affect display
PAG: periaqueductal gray
PET: positron emission tomography
PFC, bundle: prefrontal cortex to M1 (mtrMFB)
PL: pathological laughter
PP: projection pathway
RN: red nucleus
slMFB: superolateral branch of the MFB
STN: subthalamic nucleus
VAT: volume of activated tissue
VC/VS: ventral capsule ventral striatum (DBS target)
VTA: ventral tegmental area
VTG: ventral tegmental nucleus of Gudden.
